# Voluntary exercise in mice fed an obesogenic diet alters the hepatic immune phenotype and improves metabolic parameters – an animal model of life style intervention in NAFLD

**DOI:** 10.1038/s41598-018-38321-9

**Published:** 2019-03-08

**Authors:** Nadine Gehrke, Jana Biedenbach, Yvonne Huber, Beate K. Straub, Peter R. Galle, Perikles Simon, Jörn M. Schattenberg

**Affiliations:** 1 I. Department of Medicine, University Medical Center of the Johannes Gutenberg University, Mainz, Germany; 2 Institute of Pathology, University Medical Center of the Johannes Gutenberg University, Mainz, Germany; 3 Department of Sports Medicine, Rehabilitation and Prevention, Johannes Gutenberg University, Mainz, Germany

## Abstract

Reproducible animal models to recapitulate the pathophysiology of non-alcoholic fatty liver disease (NAFLD) are urgently required to improve the understanding of the mechanisms of liver injury and to explore novel therapeutic options. Current guidelines recommend life-style interventions as first-line therapy for NAFLD and these types of intervention are considered standard-of-care. The current study establishes a reproducible mouse model of a life-style intervention in NAFLD using voluntary wheel running (VWR). Male C57BL/6J mice were fed a high-fat, high-carbohydrate diet (HFD) to induce NAFLD or a corresponding control diet for 12 weeks. Starting at week 9 of the obesogenic NAFLD diet, mice were randomized to either free access to a running wheel or being single caged resembling a sedentary (SED) life-style. VWR induced a transient weight reduction in HFD-fed mice up until week 10. In contrast to the SED mice, VWR mice exhibited normal ALT at the end of the intervention, while the metabolic alterations including elevated fasting glucose, insulin, triglyceride, and total cholesterol levels remained almost unchanged. Additionally, VWR prevented HFD-induced hepatic steatosis by alterations in key liver metabolic processes including the induction of fatty acid β-oxidation and lipogenesis inhibition following increased AMP-activated protein kinase (AMPK)-α activity. Phosphorylation of the serine kinase Akt in hepatic tissue was enhanced following VWR. Furthermore, VWR mice were protected from HFD-induced expression of pro-inflammatory cytokines, chemokines and liver macrophage infiltration. The SED/HFD group exhibited increasing activity of hepatic nuclear factor (NF)-κB p65, which was absent following exercise in the VWR/HFD group. In summary, in an obesogenic mouse model of NAFLD physical exercise improves fatty acid and glucose homeostasis and protects from macrophage-associated hepatic inflammation.

## Introduction

Non-alcoholic fatty liver disease (NAFLD) is the most frequent liver disease at a global scale and increasing numbers are expected in Europe^1^. This increase is linked to the epidemic of obesity, which arises in the context of intrinsic and extrinsic risk factors, including the type of diet and lifestyle, but also genetic aspects^2^. NAFLD can progress from hepatic steatosis to inflammatory non-alcoholic steatohepatitis (NASH), which promotes the development of hepatic fibrosis leading to end-stage liver disease or even hepatocellular carcinoma (HCC). In addition, patients with NAFLD exhibit systemic, metabolic inflammation and are at higher risk of cardiovascular disease or stroke^3^. Early treatment of NAFLD is crucial in order to prevent disease progression and associated liver-related and cardio-vascular mortality. Current US and EU-guidelines recommend weight loss through dietary changes and physical exercise^4,5^. This is supported by a series of clinical studies in humans showing a reversal of steatohepatitis and regression of fibrosis with more than 7% of weight reduction^6–8^. Interestingly, a reduction of hepatic fat content from exercise occurs even in the absence of weight loss as summarized in a recent review^9^. On the other hand, a subgroup of patients does not improve liver histology, despite weight loss^10^, indicating, that body composition is likely of importance in addition to reducing body weight. A number of animal studies have explored the beneficial effects of exercise. Using a high-fat dietary model, forced exercise was shown to decrease inflammation in the adipose and hepatic tissue^11,12^. Additionally, exercise was shown to reduce hepatic fatty acid synthesis by decreasing lipogenic enzymes and AMP-activated protein kinase (AMPK)^13,14^. Also, mitochondrial inner membrane integrity and fatty acid oxidation are improved from exercise in mice^15^. Only few studies have explored the effect of exercise training on hepatic inflammation and fibrosis. In *foz/foz* mice, which develop an obese phenotype from hyperphagia, adipose tissue inflammation, hepatic inflammation, fibrosis and muscle insulin sensitivity improved with exercise^16^. In contrast to the most commonly used forced exercise models, the effectiveness of voluntary wheel running (VWR) on the hepatic immunophenotype is less clear. We have previously assessed this type of exercise during acute liver injury^17^, but data relating to obesity-induced NAFLD is currently not available. Therefore, we explored the applicability and effectiveness of VWR compared to sedentary life-style on changes in the hepatic metabolism and immune phenotype in a well-established obesogenic high-fat, high-carbohydrate diet (HFD) model of NAFLD^18^. The aim was to provide a reproducible, well-characterized and applicable mouse model to reflect the current standard of care for NAFLD and which could be used in the context of studying the additional effects of therapeutic drug compounds in addition to life-style.

## Material and Methods

### Animal model

All animals were bred and held at the animal facility of the University Medical Center Mainz, according to the criteria outlined by the “Guide for the Care and Use of Laboratory Animals”. The study was conducted following approval by the committee for experimental animal research (Landesuntersuchungsamt Rheinland-Pfalz) and all experiments were performed in accordance with relevant guidelines and regulations. We employed an obesogenic diet in 8–10-week-old, male C57BL/6J mice by feeding a high-fat diet (35.5% w/w crude fat (58 kJ%), 22.8 MJ/kg = 5.45 kcal/g) and fructose (55% w/v) and glucose (45% w/v) enriched drinking water. Gender- and age-matched controls received a matched control diet (CD; 5.4% w/w crude fat (13 kJ%), 15.7 MJ/kg = 3.74 kcal/g) and plain water^18^. The composition and energy density of the diets (ssniff Spezialdiäten GmbH, Soest, Germany) are listed in Suppl. Table 1. After 8 weeks of dietary feeding, mice were randomly assigned to a voluntary wheel running (VWR) group or a sedentary (SED) group. The VWR mice (n = 8 mice on HFD and n = 7 mice on CD) were individually housed in cages (size 43 cm in length × 25 cm width and 28 cm height) and outfitted with a 11.5 cm diameter running wheel (Suppl. Fig. 1). Wheel running activity was continuously recorded using a usual bicycle tachometer (Ciclosport, Krailling, Germany). The SED mice (n = 20 mice on HFD and n = 11 mice on CD) were housed in single in corresponding cages without running wheel. The experimental setup is shown in Suppl. Fig. 2. For the duration of the study, all mice were kept on a 12-h light/dark cycle at constant temperature (22 ± 2 °C) and humidity (55 ± 10%) and with free access to the experimental diets and water. Biometric data including body weight and food consumption were measured weekly. After the 12-week experimental period, all mice were kept sedentary and were fasted overnight before sacrifice for collection of blood and liver tissue.

### Serological analysis

Serum was obtained from 16–18-h fasted, anesthetized mice by retro-orbital bleeding (at week 8) or by cardiac puncture (at week 12) and assayed for levels of alanine-aminotransferase (ALT), glucose, total cholesterol and triglycerides using a standard clinical analyzer (Hitachi 917; Roche, Mannheim, Germany). Insulin was measured using the Rat/Mouse Insulin ELISA Kit (EMD Millipore, St. Charles, MO, USA). Monocyte-chemoattractant protein-1 (MCP-1/CCL2) levels in serum were determined by BD Cytometric Bead Array (BD Biosciences, San Diego, CA, USA).

### Histological analysis

For histological evaluation, representative liver sections were cut, fixed in 4% paraformaldehyde-PBS, embedded in paraffin, and stained with hematoxylin and eosin (H&E) following standard procedures. Semiquantitative evaluation of steatosis, inflammation and ballooning in liver tissue from at least 4–8 mice per group was done blinded by an experienced histopathologist (BKS) according to the non-alcoholic activity score (NAS) established by Kleiner and coauthors^19^. Representative pictures were obtained using an Olympus BX45 microscope (Olympus Deutschland, Hamburg, Germany) with a Jenoptik PROGRES GRYPHAX camera (Micro Optimal, Kirchheim/Teck, Meerbusch, Germany) and the Olympus Image Analysis software analySIS docu (Olympus Deutschland).

### Determination of the hepatic triglyceride content

Hepatic triglycerides were measured using the Triglyceride Quantification Kit according to the manufacturer’s instructions (BioVision, Milpitas, CA, USA).

### Quantitative real-time PCR

Isolation of total RNA, cDNA synthesis and quantitative real-time PCR (qRT-PCR) were performed as previously described^20^. All samples were performed in duplicate. Roche LightCycler software (LightCycler 480 Software Release 1.5.0, Roche, Mannheim, Germany) was used to perform advanced analysis relative quantification using the 2^(−ΔΔC(T))^ method. Expression data were normalized to the housekeeping gene *Gapdh* (primers from Qiagen, Hilden, Germany) and the mean of SED/CD mice was considered 1. Primer sequences (all primers obtained from Eurofins Genomics, Ebersberg, Germany) are listed in Suppl. Table 2.

### Immunoblotting and determination of nuclear factor (NF)-κB p65 DNA binding activity

Proteins were isolated and separated as previously described^19^. Primary antibodies included: AMPK-α, phospho-AMPK-α (Thr172), Akt, phospho-Akt (Ser473), NF-κB p65, and phospho-NF-κB p65 (Ser536) (all obtained from Cell Signaling Technology, Danvers, MA, USA). Membranes were exposed to anti-rabbit secondary antibodies conjugated with horseradish peroxidase (Santa Cruz Biotechnology, Dallas, TX, USA). PaperPort Professional software v14.0 (Nuance Communications Germany, München, Germany) was used for image acquisition and the Adobe Acrobat Professional software program (Adobe Systems Incorporated, San Jose, CA, USA) was used to cut immunoblot images to size. No post-processing of images was performed and original uncut images blots are provided in Suppl. Figs. 3 and 4. Densitometry was performed using the National Institutes of Health ImageJ software. NF-κB p65 activity was measured in duplicate in whole liver tissue using the TransAM NF-κB Family Kit, according the manufacturer’s instructions (Active Motif, Carlsbad, CA, USA) and the mean of SED/CD mice was considered 1.

### Flow cytometric analysis of intrahepatic immune cells

Isolation of intrahepatic immune cells and their flow cytometric analysis were performed as previously described^21^. Quantification of the macrophage population was performed by gating on living CD45^+^F4/80^+^ cells (all antibodies obtained from BioLegend, San Diego, CA, USA).

### Statistical analysis

All statistical analyses were performed using GraphPad Prism 7 software (GraphPad Software, La Jolla, CA, USA). All results were initially submitted to Shapiro-Wilk normality test for normality and to Levene’s test for homogeneity of variance. Comparisons between experimental groups were carried out using the unpaired two-tailed Student’s *t* test or the Mann-Whitney *U* test to determine statistical significance of differences. Results with a *p* value of <0.05 were considered to be significant. The significance-level α was adjusted using Holm’s sequential Bonferroni adjustment in analyses involving multiple comparisons. All data are shown as mean ± standard error of mean (SEM) to determine the precision and differences of means and statistically significant values were assumed with *^/$^p < 0.05, **^/$$^p < 0.01, ***^/$$$^p < 0.001.

## Results

### Impact of physical exercise on body weight and food uptake

**Figure 1:**
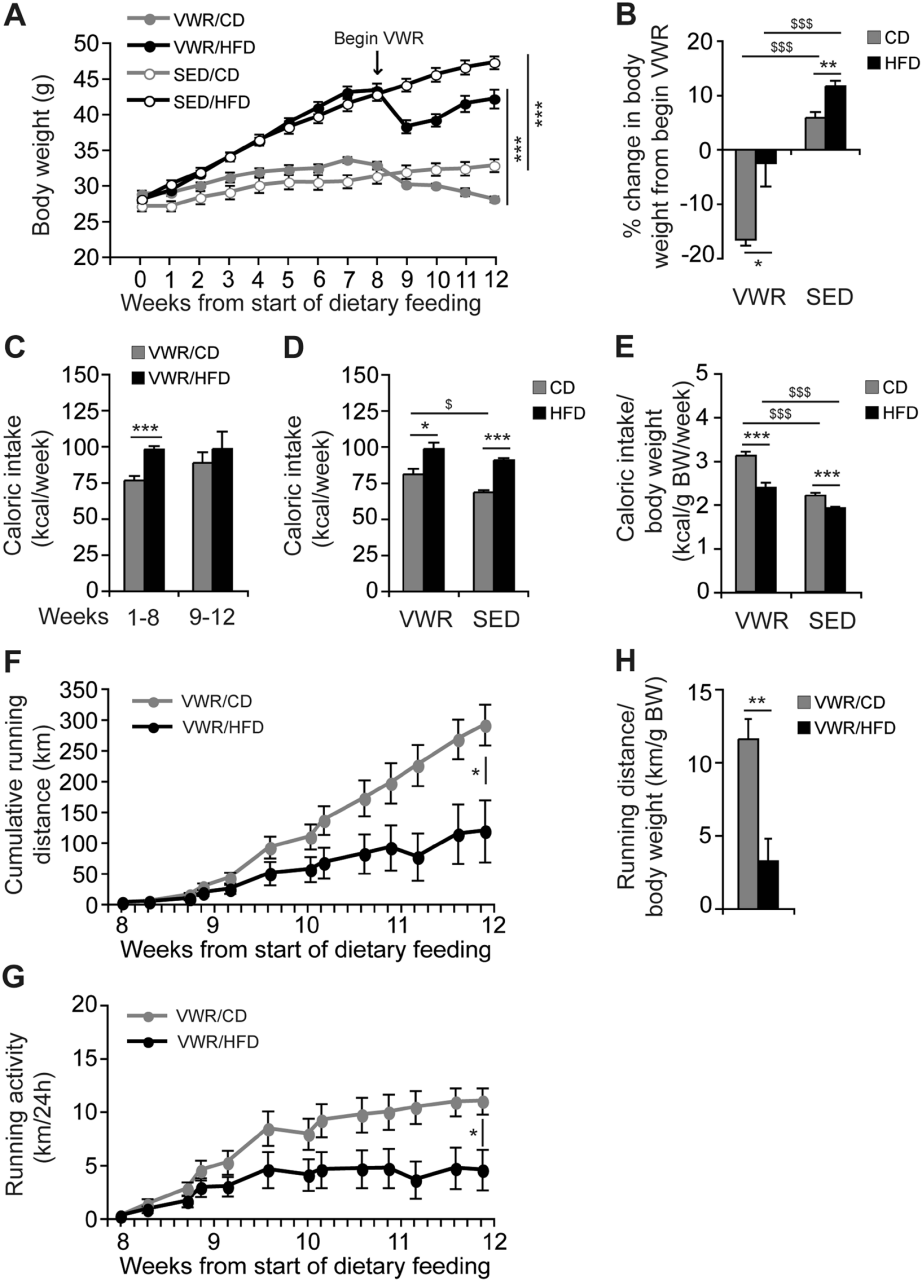
The effects of diet and running wheel access on body weight, caloric intake and physical activity. C57BL/6J mice received a high-fat, high-carbohydrate diet (HFD) or corresponding control diet (CD) for 12 weeks. After 8 weeks of dietary feeding, mice were randomly assigned to either a group given access to voluntary running wheels (VWR) for 4 weeks or a sedentary control group (SED). (**A**) Body weight curve of the VWR/CD, VWR/HFD, SED/CD, and SED/HFD group was monitored and (**B**) % change in body weight in the VWR groups during the 4 week-exercise period was compared with diet-matched SED groups. (**C**) Caloric intake of the VWR/CD and VWR/HFD group during the pre-exercise, during-exercise and (**D**) whole experimental period compared with SED controls and (**E**) ratio of average caloric intake to final body weight for all experimental groups. (**F**) Cumulative running distance, (**G**) average daily running distance, and (**H**) ratio of total running distance to final body weight for mice fed the CD or HFD. Data represent means ± SEM of (**A–H**) n = 7 VWR/CD, (**A–H**) n = 8 VWR/HFD, (**A**,**B**,**D** and **E**) n = 11 SED/CD and (**A**,**B**,**D** and **E**) n = 20 SED/HFD mice. *p < 0.05, **p < 0.01, ***p < 0.001 for CD vs. HFD and ^$^p < 0.05, ^$$$^p < 0.001 for SED vs. VWR using Mann-Whitney *U* test (**A,F–H**) and two-tailed Student’s *t*-test (**B**–**E**).

HFD-feeding induced a significant weight gain in mice compared to mice on a corresponding CD up to week 8 (Fig. 1A). After randomization to either VWR or SED at week 8, mice were continued on either CD or HFD ad libitum. For final analysis four groups were compared: VWR/CD, VWR/HFD, SED/CD and SED/HFD. All mice in the VWR/HFD group exhibited a transient weight loss until week 10, while body weight increased again up until week 12 (Fig. 1A). All mice on HFD exhibited a significantly higher body weight compared to CD-fed mice at the end of the study (SED/CD (n = 11): 31.4 ± 1.0 g vs. SED/HFD (n = 20): 47.5 ± 1.0 g, p < 0.001 and VWR/CD (n = 7): 26.2 ± 0.5 g vs. VWR/HFD (n = 8): 41.8 ± 1.5 g, p < 0.001; Fig. 1A). The relative weight change compared to baseline was significantly higher in the SED compared to the diet-matched VWR group (SED/CD (n = 11): 25.7 ± 2.0% vs. VWR/CD (n = 7): −2.9 ± 1.6%, p < 0.001 using two-tailed Student’s *t*-test; SED/HFD (n = 20): 82.4 ± 3.8% vs. VWR/HFD (n = 8): 60.2 ± 3.8%, p < 0.01 using two-tailed Student’s *t*-test). Comparing SED and VWR groups on HFD during the 4 week-exercise period, the weight gain was significantly higher in inactive mice (SED/HFD (n = 20): 11.9 ± 1.0% vs. VWR/HFD (n = 8): −2.5 ± 4.4%, p < 0.001, Fig. 1B). In the CD group, sedentary mice exhibited a 6.0 ± 1.1% increased of body weight, while active mice decreased weight by 16.8 ± 1.1% (SED/CD (n = 11) vs. VWR/CD (n = 7): p < 0.001).

Calorie uptake derived from the diet was higher in mice on HFD compared to the CD group (Fig. 1C,D). Interestingly, the VWR group on CD, but not HFD further increased calorie uptake (SED/CD (n = 11) 68.5 ± 1.6 kcal/week vs. VWR/CD (n = 7) 81.0 ± 3.7 kcal/week, p < 0.05; SED/HFD (n = 20): 90.7 ± 1.5 kcal/week vs. VWR/HFD (n = 8) 98.6 ± 4.3 kcal/week, p = ns, Fig. 1D). When examining energy uptake relative to body weight, consumed calories/g body weight were higher in the VWR groups (SED/CD (n = 11): 2.2 ± 0.1 kcal/g BW per week vs. VWR/CD (n = 7): 3.1 ± 0.1 kcal/g BW per week, p < 0.001; SED/HFD (n = 20): 1.9 ± 0.0 kcal/week vs. VWR/HFD (n = 8): 2.4 ± 0.1 kcal/g BW per week, p < 0.001, Fig. 1E).

Activity was monitored using a calibrated cycling computer connected to the exercise wheel (see Suppl. Fig. 1). Interestingly, physical activity was significantly lower in the HFD group as evident by a lower cumulative running distance over 4-weeks (VWR/HFD (n = 8): 121.0 ± 52.8 km vs. VWR/CD (n = 7): 301.0 ± 34.6 km, p < 0.05, Fig. 1F), as well as average daily distance (VWR/HFD (n = 8): 4.5 ± 2.0 km/d vs. VWR/CD (n = 7): 11.1 ± 1.3 km/d, p < 0.05, Fig. 1G). In relation to body weight, the running distance was lower in the VWR/HFD group (VWR/HFD (n = 8): 3.3 ± 1.6 km/g BW vs. VWR/CD (n = 7): 11.6 ± 1.4 km/g BW, p < 0.01, Fig. 1H). At an individual level, a large variety of running activity was detected ranging from 9.3 to 454.4 km in the VWR/HFD and 274.9 to 371.7 km in the VWR/CD group over 4 weeks. Distance was independent of starting body weight.

### Effects of physical exercise on the metabolic phenotype

**Figure 2:**
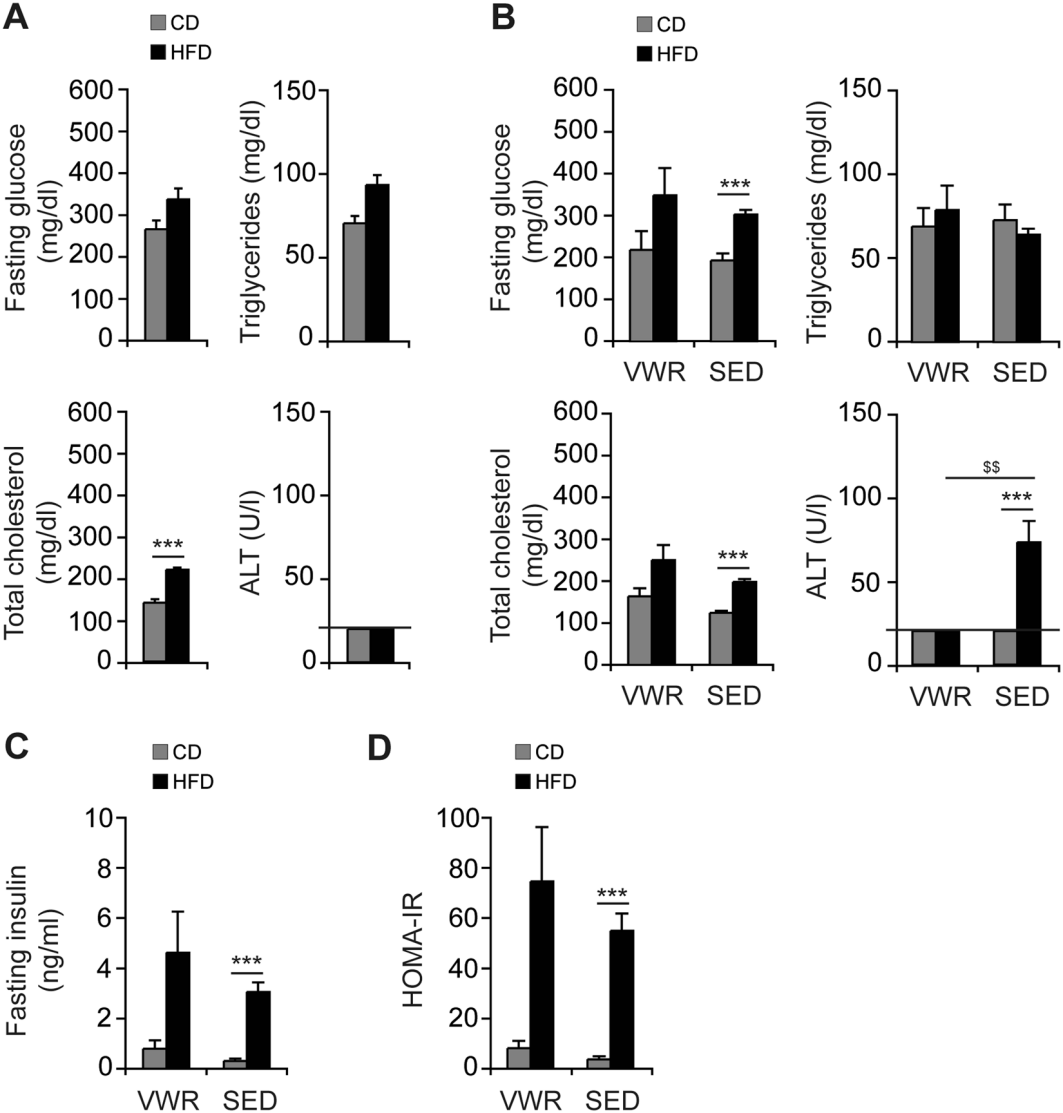
Effects of VWR on liver enzymes and metabolic parameters. Fasting levels of glucose, triglycerides, total cholesterol and ALT of CD- and HFD-fed mice were assessed (**A**) pre-exercise and (**B**) post-exercise in comparison with diet-matched SED groups. (**C**) Fasting insulin levels and (**D**) HOMA-IR (fasting insulin concentration (pmol/l) x fasting glucose concentration (mmol/l)/22.5) in the VWR and SED groups after 12 weeks of feeding. Data are means ± SEM of (**A**–**D**) n = 7 VWR/CD, (**A**–**D**) n = 8 VWR/HFD, (**B**–**D**) n = 11 SED/CD and (**B**–**D**) n = 20 SED/HFD mice. ***p < 0.001 for CD vs. HFD and ^$$^p < 0.01 for SED vs. VWR using two-tailed Student’s *t*-test (**A** and **B** (fasting glucose, total cholesterol)) or Mann-Whitney *U* test (**B** (ALT)-**D**).

The development of HFD-induced obesity at week 8 was associated with increased total cholesterol (HFD (n = 8): 221.1 ± 5.3 mg/dl vs. CD (n = 7): 141.4 ± 8.6 mg/dl, p < 0.001), while no changes in fasting glucose (HFD (n = 8): 336.0 ± 26.2 mg/dl vs. CD (n = 7): 264.9 ± 21.3 mg/dl, p = ns), or triglycerides (HFD (n = 8): 93.1 ± 6.2 mg/dl vs. CD (n = 7): 78.9 ± 4.4 mg/dl, p = ns) were detected (Fig. 2A). At week 12 fasting glucose levels and total cholesterol levels were elevated in the HFD compared to CD-fed controls (SED/HFD (n = 20) vs. SED/CD (n = 11): 300.8 ± 10.6 mg/dl vs. 191.3 ± 17.4 mg/dl fasting glucose, p < 0.001; 197.8 ± 6.6 mg/dl vs. 123.6 ± 4.9 mg/dl total cholesterol, p < 0.001) but not triglycerides (64.4 ± 3.6 mg/dl vs. 73.1 ± 9.3 mg/dl, p = ns). Physical exercise did not correct hyperglycemia or total cholesterol and there were no statistically significant differences in these metabolic parameters between the VWR/HFD and SED/HFD group (Fig. 2B). Moreover, physical exercise did not correct fasting insulin levels in HFD-fed mice (VWR/HFD (n = 8): 4.6 ± 1.6 ng/ml vs. SED/HFD (n = 20): 3.1 ± 0.4 ng/ml, p = ns, Fig. 2C). Hence insulin resistance defined by HOMA-IR index, which was significantly higher in the SED/HFD group compared to the SED/CD group (SED/HFD (n = 20): 55.5 ± 7.0 vs. SED/CD (n = 11): 3.8 ± 1.2, p < 0.001, Fig. 2D), remained largely unaffected in this physical exercise model. Mice on CD exhibited stable metabolic profiles, including fasting glucose, insulin, triglycerides and total cholesterol throughout the entire feeding and exercise period. HFD-feeding increased ALT in SED mice (SED/HFD (n = 20): 73.00 ± 12.7 U/l vs. SED/CD (n = 11): <20.0 U/l, p < 0.001) at 12 weeks (Fig. 2B), while ALT was not elevated in the VWR/HFD, SED/CD or VWR/CD mice (all <20.0 U/l, Fig. 2A,B).

### Effects of physical exercise on hepatic steatosis and lipid metabolism

**Figure 3:**
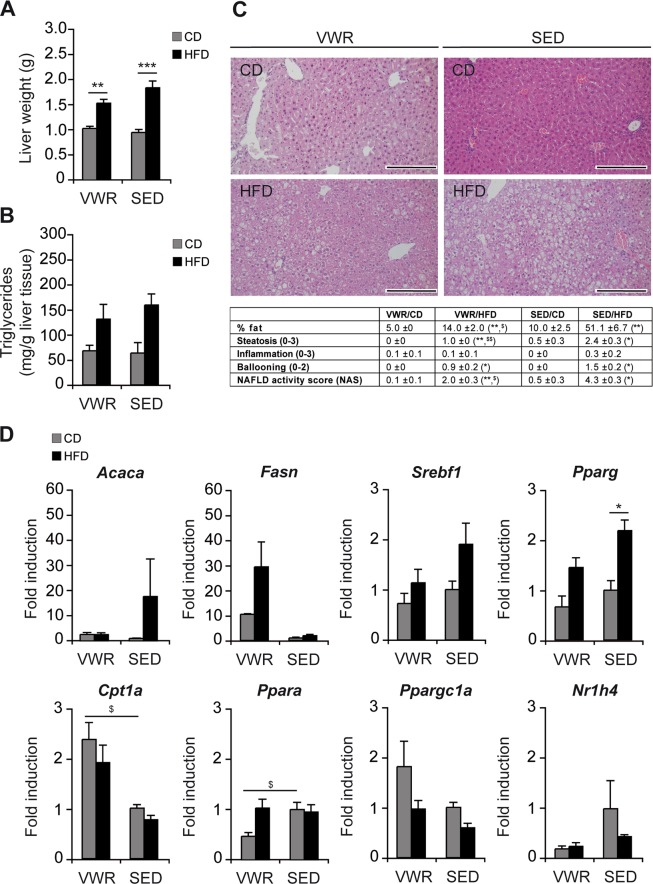
Effects of VWR on hepatic lipid metabolism and steatosis in HFD-fed mice. (**A**) Absolute liver weight, (**B**) liver triglyceride content, (**C**) representative liver histology by H&E staining (scale bar: 200 µm) and NAFLD activity score (NAS), and (**D**) relative mRNA expression of key regulators of hepatic lipid metabolism determined by qRT-PCR in VWR and SED mice after 12 weeks of dietary feeding. Data represent means ± SEM of (**A**) n = 7 VWR/CD, n = 8 VWR/HFD, n = 11 SED/CD, n = 20 SED/HFD, (**B**) n = 5 mice/group, (**C**) n = 7 VWR/CD, n = 8 VWR/HFD, n = 4 SED/CD, n = 8 SED/HFD, (**D**) n = 7 VWR/CD, n = 8 VWR/HFD, n = 5 SED/CD and n = 5 SED/HFD mice. *p < 0.05, **p < 0.01, ***p < 0.001 for CD vs. HFD and ^$^p < 0.05, ^$$^p < 0.01 for SED vs. VWR using Mann-Whitney *U* test (**A**,**C**) or two-tailed Student’s *t*-test (**D**).

Mice on HFD developed hepatomegaly by week 12 irrespective of exercise (liver weight: VWR/HFD (n = 8): 1.53 ± 0.08 g vs. VWR/CD (n = 7): 1.03 ± 0.04 g, p < 0.01, Fig. 3A). Quantification of intrahepatic triglycerides showed comparable amounts between the groups (hepatic triglycerides/g liver tissue: VWR/HFD (n = 5): 131.4 ± 29.3 mg/g, SED/HFD (n = 5): 159.5 ± 22.2 mg/g, VWR/CD (n = 5): 68.3 ± 10.8 mg/g, SED/CD (n = 5): 63.5 ± 20.8 mg/g, Fig. 3B). Histological evaluation of H&E-stained liver sections showed macrovesicular steatosis with few ballooned hepatocytes and minimal lobular inflammation in the SED/HFD group, while there was a significantly lower degree of hepatic steatosis in the VWR/HFD group (VWR/HFD (n = 8): 14.0 ± 2.0% fat vs. SED/HFD (n = 8): 51.1 ± 6.7% fat, p < 0.05, Fig. 3C). While hepatocyte ballooning and inflammation were not significantly reduced following exercise, the NAS score decreased significantly (VWR/HFD (n = 8): 2.0 ± 0.3 vs. SED/HFD (n = 8): 4.3 ± 0.2, p < 0.05, Fig. 3C). These data suggest that the 4-week VWR-based exercise training was an effective intervention to improve the histological hallmarks – predominantly hepatic steatosis – of NAFLD.

To investigate the molecular effects of exercise, fatty acid metabolism-associated genes were analyzed using qRT-PCR (Fig. 3D). Hepatic gene expression of lipogenic enzymes and associated transcription factors including acetyl-CoA carboxylase (ACC), fatty acid synthase (FAS), sterol regulatory element binding protein-1c (SREBP-1c) and peroxisome proliferator-activated receptor (PPAR)-γ *–* encoded by *Acaca*, *Fasn*, *Srebf1* and *Pparg –* were examined and significant changes from 12 weeks of HFD feeding were observed only for Pparg (*Acaca*: SED/HFD (n = 5): 17.7 ± 15.0 -fold vs. SED/CD (n = 5): 1.0 ± 0.1 -fold, p = ns, *Fasn*: SED/HFD (n = 5): 1.9 ± 0.5 -fold vs. SED/CD (n = 5): 1.0 ± 0.3, p = ns, *Srebf1*: SED/HFD (n = 5): 1.9 ± 0.4 -fold vs. SED/CD (n = 5): 1.0 ± 0.2 -fold, p = ns, *Pparg*: SED/HFD (n = 5): 2.2 ± 0.2 -fold vs. SED/CD (n = 5): 1.0 ± 0.2 -fold, p < 0.05). Physical exercise did not result in changes of HFD-associated expression of ACC, SREBP-1c, PPAR-γ, or FAS. Analysis of genes involved in mitochondrial fatty acid β-oxidation showed that hepatic PPAR-α expression was not affected by HFD and comparable in VWR and SED mice, but significantly downregulated in the VWR/CD group (VWR/CD (n = 7): 0.5 ± 0.1 -fold vs. SED/CD (n = 5): 1.0 ± 0.1 -fold, p < 0.05). By contrast, levels of carnitine palmitoyltransferase I (CPT1) - encoded by *Cpt1a* - were significantly upregulated only in CD-diet fed mice from exercise (VWR/CD (n = 7): 2.4 ± 0.3-fold vs. SED/CD (n = 5): 1.0 ± 0.1-fold, p < 0.05,) while this effect was blunted in the HFD-group (VWR/HFD (n = 8): 1.9 ± 0.3-fold vs. SED/HFD (n = 5): 0.8 ± 0.1-fold, p = ns). On the other hand, *Ppargc1a* encoding peroxisome proliferator-activated receptor-gamma coactivator-1α (PGC-1α) was not significantly different(VWR/CD (n = 7): 1.8 ± 0.5-fold vs. SED/CD (n = 5): 1.0 ± 0.1, p = ns, VWR/HFD (n = 8): 1.0 ± 0.2 -fold vs. SED/HFD (n = 5): 0.6 ± 0.1-fold, p = ns). The expression of the farnesoid X receptor (FXR)-α, which is encoded by *Nr1h4*, or FXR-β (data not shown) were not significantly different between the groups, suggesting that PGC-1α acts independently from FXR-α/β.

### AMPK and Akt signaling in obesity and exercise

**Figure 4:**
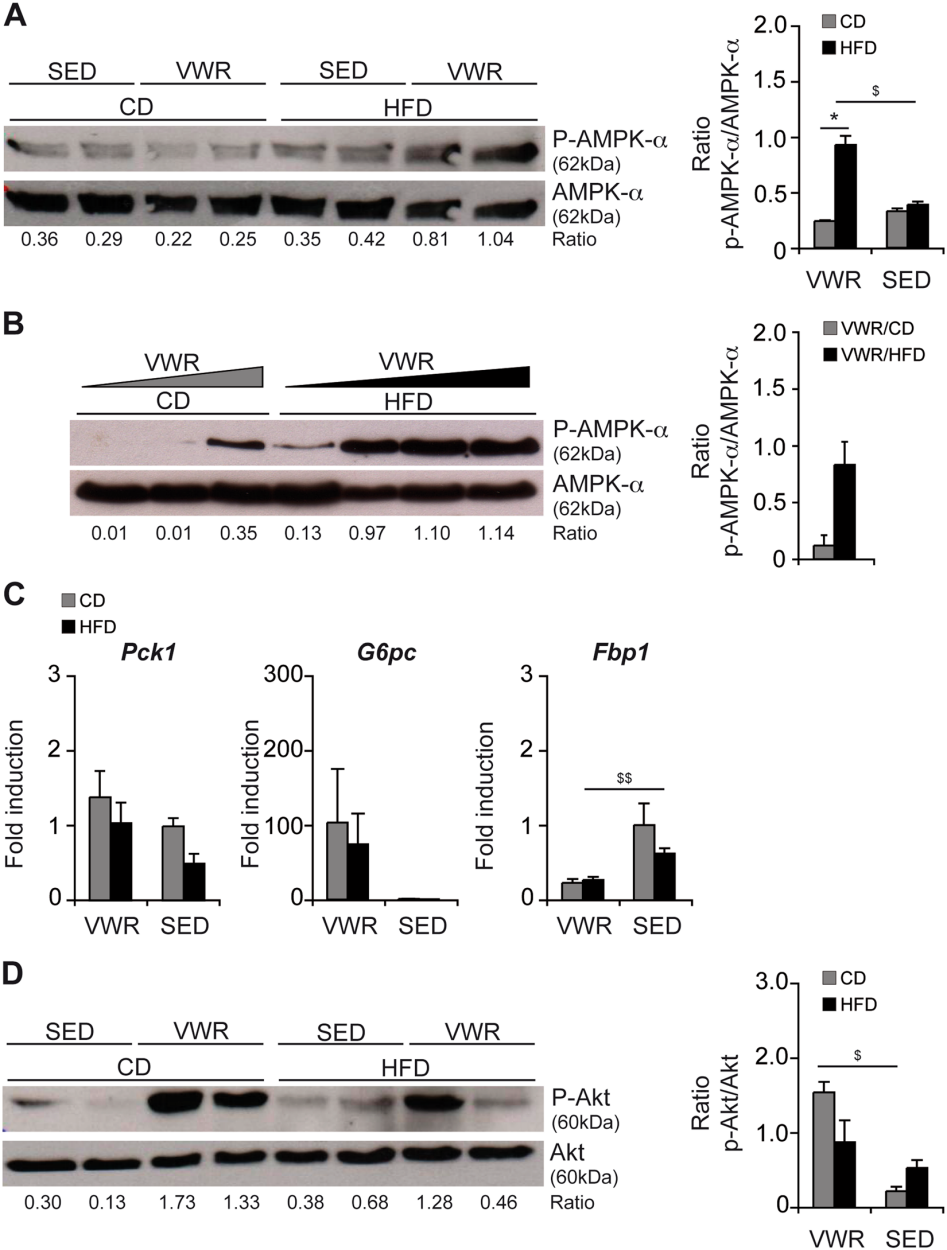
Effects of VWR on AMPK-α activation and glucose metabolism in the liver. (**A**) Western blot and densitometric analyses of phospho-AMPK-α-Thr172 and total AMPK-α protein in liver lysates from VWR vs. SED mice fed the diets for 12 weeks and (**B**) from VWR mice on CD or HFD with increasing individual physical activity levels (increasing from left to right). (**C**) qRT-PCR analyses of gluconeogenetic enzymes and (**D**) Western blot with densitometric analysis of phosphorylated (Ser473) and total Akt protein in the liver of the different experimental groups after 12 weeks of feeding. In A, B and D representative immunoblots with densitometric analysis are shown. Uncropped images of original blots are shown in Suppl. Fig. 3A–C. Data in C represent means ± SEM of n = 7 VWR/CD, n = 8 VWR/HFD, n = 5 SED/CD and n = 5 SED/HFD mice. *p < 0.05 for CD vs. HFD and ^$^p < 0.05, ^$$^p < 0.01 for SED vs. VWR using two-tailed Student’s *t*-test (**A**–**D**).

Physical activity is a powerful activator of AMPK, a key regulator of cellular energy homeostasis. In the employed exercise model, activation of AMPK-α – detected by phosphorylation at Thr172 residue – was observed in VWR/HFD mice (densitometry ratio: p-AMPK-α/AMPK-α: VWR/HFD (n = 2): 0.93 ± 0.08 vs. VWR/CD (n = 2): 0.24 ± 0.01, p < 0.05, VWR/HFD vs. SED/HFD (n = 2): 0.39 ± 0.02, p < 0.05, Fig. 4A). The levels of physical activity and AMPK-α phosphorylation exhibited a positive correlation when comparing mice with a low vs. high running distance (Fig. 4B). In parallel, we observed a slight reduction of total AMPK-α in liver tissue from exercise (Fig. 4A,B).

Physical activity altered the expression of the gluconeogenic enzymes phosphoenolpyruvate carboxykinase (PEPCK) and glucose-6 phosphatase (G6Pase) - encoded by *Pck1* and *G6pc* - irrespective of the underlying diet (Fig. 4C), which both are regulators of insulin sensitivity^22^. The differences between VWR and diet-matched SED groups were not significant (Fig. 4C). Fructose-1,6-bisphosphatase 1 (FBP1) – encoded by *Fbp1* – was decreased in expression from exercise following HFD-feeding but not CD-feeding (VWR/CD (n = 7): 0.2 ± 0.1 vs. SED/CD (n = 5): 1.0 ± 0.3, p = ns, VWR/HFD (n = 8): 0.3 ± 0.0 vs. SED/HFD (n = 5): 0.6 ± 0.1, p < 0.01, Fig. 4C).

The serine-threonine protein kinase B (PKB/Akt) is activated by serine phosphorylation and controls hepatic glucose metabolism^23^. Densitometry of immunoblots showed increased levels of Ser473-phosphorylation in mice of the exercise groups (Fig. 4D), while no difference was detectable when comparing VWR/HFD and VWR/CD mice (p = ns). Levels of total Akt protein were unchanged (Fig. 4D).

### Physical exercise alters the hepatic immunophenotype in an obesogenic mouse model

**Figure 5:**
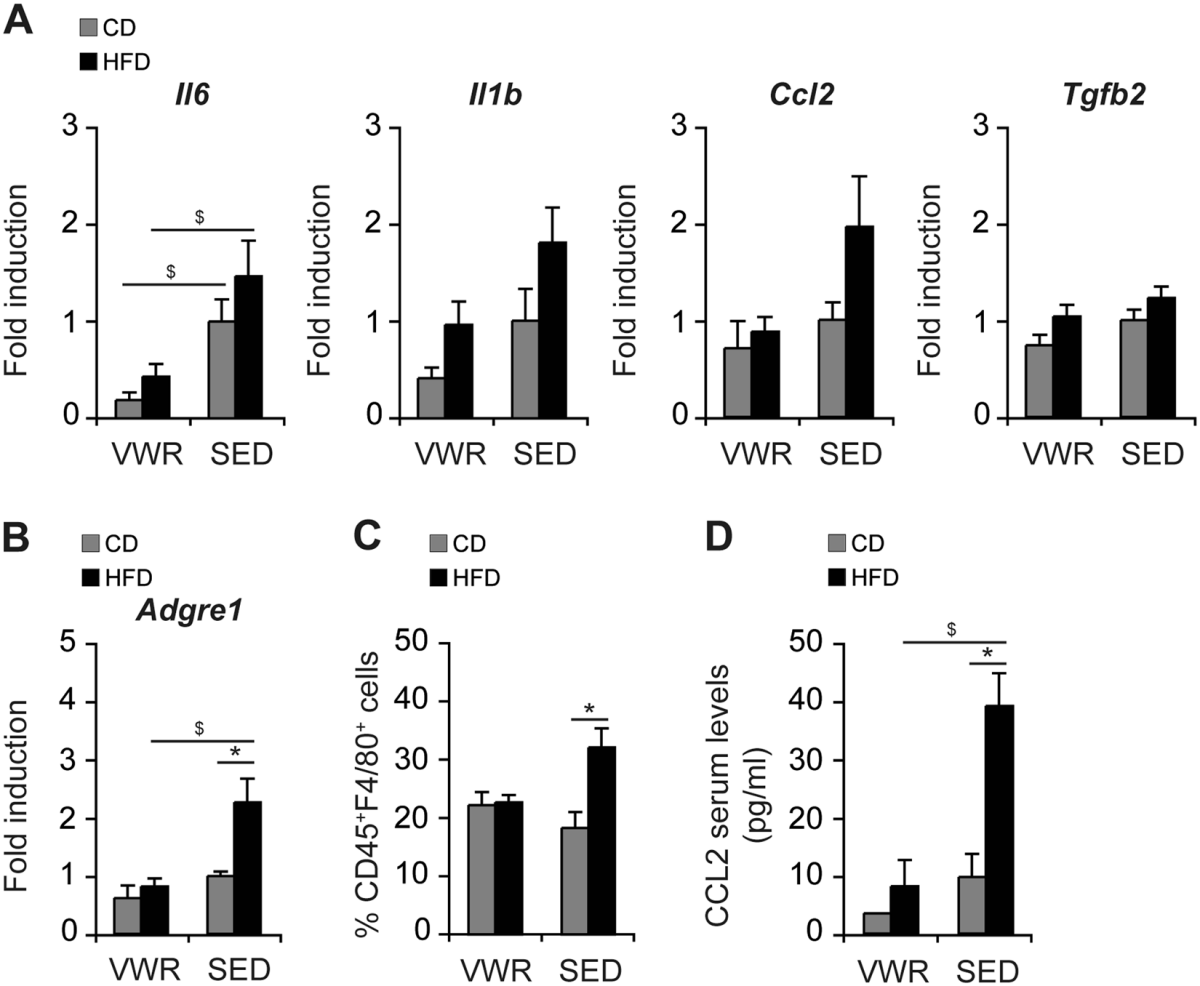
Impact of VWR on HFD-induced hepatic inflammation and macrophage infiltration. (**A**) Relative mRNA expression levels in whole liver of pro-inflammatory and pro-fibrotic cytokines, and (**B**) the macrophage-marker F4/80, (**C**) relative numbers of living, intrahepatic CD45^+^F4/80^+^ cells quantified by flow cytometry, and (**D**) CCL2 serum levels in VWR and SED mice after 12 weeks of exposure to CD or HFD. Data in A and B are means ± SEM of (**A**,**B**) n = 7 VWR/CD, n = 8 VWR/HFD, n = 5 SED/CD, n = 5 SED/HFD mice, (**C**,**D**) n = 5 VWR/CD, n = 5 VWR/HFD, n = 5 SED/CD and n = 15 SED/HFD mice. *p < 0.05 for CD vs. HFD and ^$^p < 0.05 for SED vs. VWR using Mann-Whitney *U* test (**A**,**C**) or two-tailed Student’s *t*-test (**B,D**).

Hepatic inflammation is the hallmark of progressive NAFLD. Intrahepatic pro-inflammatory cytokines were assayed by qRT-PCR-based gene expression analyses. 12-week HFD-feeding induced an increased expression of interleukin (IL)-6, IL-1β, MCP-1/CCL2 and the pro-fibrotic transforming growth factor (TGF)-β in liver tissue. Of these, IL-6 was significantly diminished from physical exercise (VWR/HFD (n = 8) vs. SEDHFD (n = 5): *Il6*: 71% (p < 0.05)) while the others were not (%reduction: *Il1b*: 47%, *Ccl2*: 55%, *Tgf2b*: 15%, Fig. 5A). Likewise, expression of the macrophage marker F4/80 - encoded by *Adgre1* - which was significantly increased in SED mice from HFD, was lowered by 64% in the VWR/HFD group from exercise (p < 0.05, Fig. 5B). To validate these results, the intrahepatic immunophenotype was assessed by FACS analysis. HFD-feeding promoted macrophage infiltration into the hepatic tissue, which was abrogated in the VWR/HFD group (Fig. 5C). While SED/HFD mice exhibited a significant increase in the relative number of intrahepatic CD45^+^F4/80^+^ cells compared to SED controls (SED/HFD (n = 15): 32.2 ± 3.3% vs. SED/CD (n = 5): 18.4 ± 2.7%, p < 0.05), the relative proportion of macrophages remained unchanged in the liver of VWR mice regardless of the type of diet (VWR/HFD (n = 5): 22.8 ± 1.2% vs. VWR/CD (n = 5): 22.3 ± 2.2%, p = ns). Additionally, HFD-induced recruitment of macrophages into the liver of SED mice was accompanied by elevated serum levels of CCL2 (SED/HFD (n = 15): 39.2 ± 5.6 pg/ml vs. SED/CD (n = 5): 9.8 ± 3.9 pg/ml, p < 0.05), which were reduced from exercise (VWR/HFD (n = 5): 8.2 ± 4.5 pg/ml vs. VWR/CD (n = 5): 3.5 ± 0 pg/ml, p = ns; SED/HFD vs. VWR/HFD: p < 0.05, Fig. 5D).

**Figure 6:**
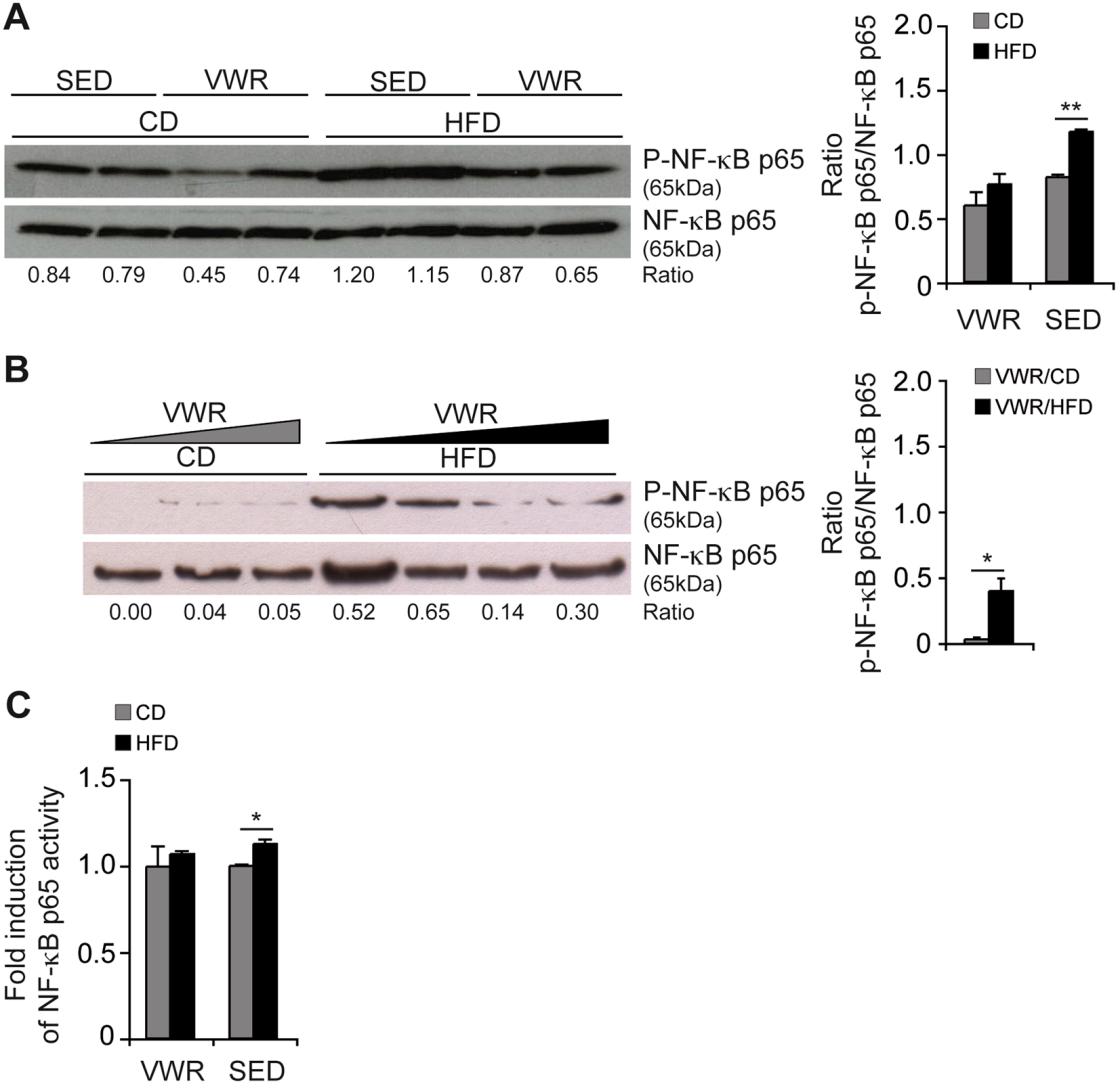
Effects of VWR on NF-κB activation in the liver of HFD-fed mice. (**A**) Phospho-NF-κB p65 (Ser536) and total NF-κB p65 protein expression were determined in liver lysates from VWR mice compared with SED mice and (**B**) from VWR mice with increasing individual physical activity levels (increasing from left to right) fed the diets for 12 weeks by western blot, and (**C**) activated p65 was quantified by a specific DNA-binding ELISA. Data are shown as fold change relative to the mean of SED/CD mice, which was considered 1. In (**A,B**) representative immunoblots with densitometric analysis are shown. Uncropped images of original blots are shown in Suppl. Fig. 4A,B. Data in C are means ± SEM of n = 5 mice/group. *p < 0.05, **p < 0.01 for CD vs. HFD using two-tailed Student’s *t*-test (**A**–**C**).

A central mediator of hepatic inflammation is NF-κB and its activity was assayed by examining phosphorylation of hepatic NF-κB p65. HFD-intake significantly enhanced NF-κB p65 phosphorylation in SED mice, while this was prevented by physical activity (Fig. 6A). Densitometry ratio analysis supported a trend with p-NF-κB p65/NF-κB p65: SED/HFD (n = 2): 1.17 ± 0.02 vs. SED/CD (n = 2): 0.82 ± 0.02, p < 0.01; SED/HFD vs. VWR/HFD (n = 2): 0.76 ± 0.08, p = 0.06 (Fig. 6A). There was a trend of phospho-NF-κB p65 levels to decrease with increasing levels of physical activity (Fig. 6B). This was also evident in a functional DNA-binding assay which showed increased NF-κB p65 activity in SED/HFD compared to SED/CD mice (SED/HFD (n = 5): 1.127 ± 0.026 -fold vs. SED/CD (n = 5): 1.000 ± 0.009 -fold, p < 0.05) and diminishing activity from VWR (VWR/HFD (n = 5): 1.068 ± 0.017-fold vs. VWR/CD (n = 5): 0.995 ± 0.119-fold, p = ns, Fig. 6C). However, the difference in NF-κB p65 activity between the SED/HFD and VWR/HFD group was not statistically significant.

As potential mediators of exercise-induced NAFLD improvements serum adiponectin and IL-6 were determined. Serum levels of total adiponectin significantly decreased from 12-week HFD-feeding (SED/HFD (n = 10): 55.6 ± 3.6 µg/ml vs. SED/CD (n = 6): 80.4 ± 5.6 µg/ml, p < 0.05; VWR/HFD (n = 4): 60.2 ± 5.9 µg/ml vs. VWR/CD (n = 4): 118.8 ± 9.6 µg/ml, p < 0.05, Suppl. Fig. 5A). VWR significantly increased serum adiponectin in CD-fed mice (p < 0.05), whereas VWR/HFD mice did not show changes in adiponectin concentrations compared to SED controls (p = ns). Next, IL-6 was measured and we observed low levels in all groups. Overall, there were no significant changes of IL-6 levels (Suppl. Fig. 5B).

## Discussion

Physical exercise is an effective intervention and recommended as first line treatment in patients with NAFLD^5^. The underlying molecular mechanisms that contribute to the beneficial effects of physical exercise related to the hepatic phenotype are incompletely understood. In the literature alterations of immune cell populations and the metabolic phenotype have been described^8,11,12^. In the current study we explored the effects of a short, voluntary exercise protocol and combined this with a well-characterized obesogenic diet that produces NAFLD without severe steatohepatitis or fibrosis. This model well reflects the mild hepatic involvement of an obese, pre-diabetic patient that is most commonly encountered in hepatology clinics^24^. Physical exercise over 4 weeks followed by 8 weeks of an obesogenic diet slowed weight gain transiently but did not revert the metabolic syndrome and fasted glucose, insulin, triglyceride and total cholesterol remained elevated. Exercise induced resolution of histological changes – predominantly of hepatic steatosis - but also hepatocyte ballooning and inflammation. Therefore, we examined the effects of physical exercise on hepatic lipid metabolism. Firstly, we addressed changes in hepatic *de novo* lipogenesis and catabolic mitochondrial fatty acid β-oxidation. Among the key liponeogenic regulators PPAR-γ, which stimulates the expression of genes that control uptake, trafficking, and lipid synthesis of fatty acids in the liver^25^, was induced from HFD feeding and in addition significantly downregulated from exercise. On the catabolic side, CPT1 - a rate limiting enzyme for entry of long-chain fatty acid into the mitochondria and subsequent mitochondrial β-oxidation - was increased following exercise. Furthermore, HFD-feeding suppressed PGC1-α - a transcriptional regulator of PPAR-α and genes encoding enzymes of mitochondrial fatty acid β-oxidation. PGC-1α has previously been identified as a potent anti-steatotic factor induced from exercise and acts through increasing mitochondrial β-oxidation and disposing of potentially injurious lipid species^26^. Our data showed variability with exercise however no clear trend was observed. Previous findings suggested that exercise-induced PGC-1α acts to repress SREBP-1c resulting in decreased triglyceride synthesis and secretion. Among the PGC-1α mediated effects, increasing activation of the nuclear receptor FXR has been observed. FXR activation through the synthetic ligand obeticholic acid has been shown to exert anti-inflammatory and anti-steatotic effects in NASH, albeit increasing serum LDL levels^22^. In the current study we explored the expression of FXR-α which was unchanged from activity. Therefore, the addition of an FXR agonist to physical exercise could be of particular use to treat patients with NASH and activate all relevant signaling pathways.

The metabolic alterations in exercising mice were at least partially mediated through AMPK. AMPK is known to phosphorylate SREBP-1c at Ser372 residues and to inhibit proteolytic cleavage and nuclear translocation of SREBP-1c which represses hepatic lipogenesis^27^. Likewise, liver-specific activation of AMPK completely protects against hepatic steatosis in mice fed a high-fructose diet through inhibition of *de novo* fatty acid synthesis^28^. Apart from direct phosphorylation of metabolic enzymes, AMPK also has long-term effects at the transcriptional level and acts to adapt gene expression to energy demands. There is considerable data mostly derived from *in vitro* assays supporting that AMPK activation leads to increased PGC-1α expression and through this modulates the expression of several key players of mitochondrial function and glucose metabolism^29^. PGC-1α regulates gluconeogenic genes directly through coactivation of key transcription factors, including hepatocyte nuclear factor (HNF) 4α and forkhead box protein O1 (FOXO1), and indirectly involving other factors of insulin sensitivity^30^. Another possible explanation was provided by a study published recently by Hughey *et al*., which showed that AMPK-α promotes hepatic glycogenolysis, but does not influence gluconeogenesis in exercising mice^31^ – a finding consistent with the current observations. Our experimental data suggests that mitochondrial fatty acid β-oxidation in exercise trained mice increases – a mechanism by which hepatocytes provide energy supplies during exercise. In addition to liver-generated glucose, ketone bodies, which are synthesized in the liver form acetyl-CoA derived primarily from fatty acid oxidation, are an essential source of energy for extrahepatic tissues during exercise^32^. These metabolic adaptions during exercise contribute to improved hepatic lipid metabolism and glucose homeostasis in obese mice.

Second, key regulators of hepatic insulin signaling were examined. The principle downstream effector of insulin signaling in the liver is the serine-threonine protein kinase Akt, also known as protein kinase B (PKB)^33^. Akt activation mediates the inhibitory effects of insulin on gluconeogenesis and glycogenolysis, in addition to hepatic fatty acid oxidation^27,34^. Metabolic and oxidative stress from steatohepatitis deregulate Akt signaling and promote hepatic insulin resistance^35^. These deleterious effects were previously shown to be reversible from swimming^36^ and the current study recapitulates these findings using the voluntary running model suggesting, that physical exercise is capable of priming the liver for treatments that address insulin signaling at the level of the hepatocytes.

Thirdly, we explored inflammation and hepatocellular injury. Clinical trials examining the effects of physical exercise in patients with NAFLD suggested that endurance exercise reduces hepatic inflammation in obese subjects even in the absence of weight reduction^37^. The obesogenic model of NAFLD used in the current study is relatively mild and does not produce major liver injury or hepatic fibrosis^38^. Nonetheless, levels of ALT were elevated in sedentary mice on HFD. This was paralleled by increased expression of pro-inflammatory cytokines including IL-6, IL-1β, TGF-β and the chemotactic MCP-1/CCL2. The expression of these cytokines has been implicated in the progression to severe steatohepatitis and fibrosis^39,40^. Likewise, these pro-inflammatory cytokines impair hepatic insulin sensitivity by negatively interfering with insulin signaling and promote a vicious cycle by inducing more inflammatory cytokines^41^. Physical activity blunted cytokine expression – to the level seen in non-obese mice – and tuned down NF-κB p65 activity. In the literature there is extensive evidence linking AMPK to inhibition of NF-κB-mediated pro-inflammatory signaling either through direct effects or indirectly through downstream mediators including PGC-1α, which can subsequently repress the expression of inflammatory factors^42^. In the current study we extended these functional findings and explored the hepatic immunophenotype from hepatic steatosis and the role of physical activity in altering immune cell composition. This was suggested by the observation that MCP-1/CCL2 – a key immune-chemokine^43^ – is suppressed from exercise. Using FACS analysis, we were able to demonstrate an effect of exercise on intrahepatic CD45^+^F4/80^+^ macrophages which are decreasing following 4 weeks of voluntary exercise.

**Figure 7:**
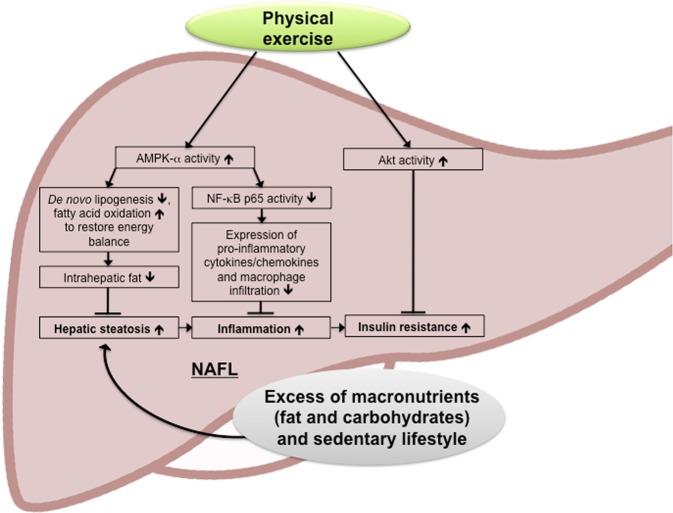
Effects of physical exercise on metabolic and inflammatory effectors in NAFLD. Physical exercise in the context of NAFLD counteracts the unfavorable steatotic, pro-inflammatory phenotype with insulin resistance by (1) increasing AMPK-α activation with beneficial effects on hepatic *de novo* lipogenesis, (2) anti-inflammatory effects evident by decreased NF-κB p65 activation as well as reduced macrophage recruitment and (3) increasing Akt activation and insulin sensitivity in the liver.

In summary, the beneficial changes of voluntary exercise – highlighted in Fig. 7 – result from improvement of the hepatic metabolism with decreasing liponeogenesis and gluconeogenesis, as well as increasing insulin sensitivity mediated through AMPK and Akt signaling. The running distance correlated with decreasing hepatic injury and improved metabolism. In addition, the inflammatory, intrahepatic immunophenotype was changed as evident by decreased expression of pro-inflammatory, chemotactic and pro-fibrogenic cytokines, reduced NF-κB signaling and dampened macrophage recruitment. In order to maximize therapeutic effects of developing pharmacological treatment, the current study highlights the effect of physical exercise that could be favorable and compensate for short comings of potential monotherapies or augment the beneficial effect of combination drug regimens.

## Conclusions

Overall, this study defines the metabolic and immunological mechanisms in a well characterized, short, voluntary exercise model combined with an obesogenic mouse model of NAFLD. Metabolic effects are in parts mediated through activation of hepatic AMPK-α and Akt. Furthermore, physical activity protects against a pro-inflammatory and pro-fibrogenic liver milieu by inhibiting the recruitment of inflammatory macrophages and thus is potentially capable of slowing the progression to NASH.

## Supplementary information

Supplementary Dataset 1

## Data Availability

All data included in the uploaded manuscript are available.
