# Supplementary material for: Voluntary exercise in mice fed an obesogenic diet alters the hepatic immune phenotype and improves metabolic parameters – an animal model of life style intervention in NAFLD
Source: Sci Rep. 2019 Mar 8;9:4007. doi: 10.1038/s41598-018-38321-9 (PMC6408519; doi:10.1038/s41598-018-38321-9)
Supplement: Supplementary file 1 — Supplementary Dataset 1 [file 41598_2018_38321_MOESM1_ESM.doc]

**Voluntary exercise in mice fed an obesogenic diet alters the hepatic immune phenotype and improves metabolic parameters – an animal model of life style intervention in NAFLD**

Nadine Gehrke^1^, Jana Biedenbach^1^, Yvonne Huber^1^, Beate K. Straub^2^, Peter R. Galle^1^, Perikles Simon^3^, Jörn M. Schattenberg^1^

^1^ I. Department of Medicine, University Medical Center of the Johannes Gutenberg University, Mainz, Germany

^2^ Institute of Pathology, University Medical Center of the Johannes Gutenberg University, Mainz, Germany

^3^ Department of Sports Medicine, Rehabilitation and Prevention, Johannes Gutenberg University, Mainz, Germany

**Table of contents:**

| **Supplementary Figures** | *Supplementary Figure 1: Voluntary wheel running in the mouse was used to examine the mechanisms by which exercise confers improvement in NAFLD.*  *Supplementary Figure 2: Experimental setup.*  *Supplementary Figure 3: Uncropped western blots represented in main Figure 4.*  *Supplementary Figure 4: Uncropped western blots represented in main Figure 6.*  *Supplementary Figure 5: Serum adiponectin and IL-6.* | Pages 2-6 |
| --- | --- | --- |
| **Supplementary Tables** | *Supplementary Table 1: Crude nutrients and energy density of experimental diets (both ssniff Spezialdiäten GmbH, Soest, Germany).*  *Supplementary Table 2: Forward and reverse primers used for qRT-PCR.* | Pages 7-8 |

**Supplementary Figures:**

*Supplementary Figure 1: Voluntary wheel running (VWR) was used to examine the mechanisms by which exercise confers improvement in NAFLD.*

**
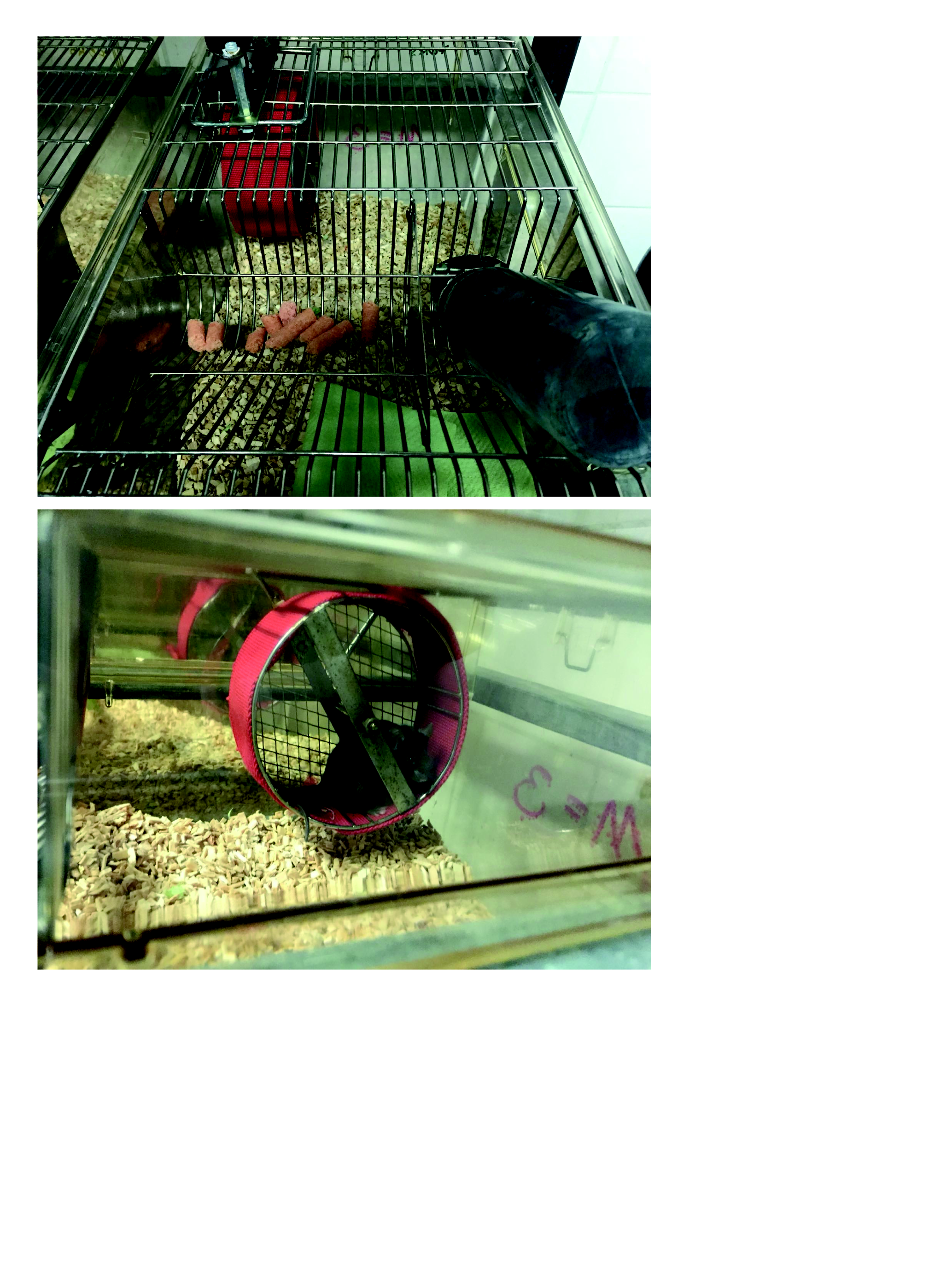
**

To study the effects of voluntary exercise, mice were housed individually in cages equipped with a running wheel. Activity was continuously monitored using a bicycle tachometer. This system allowed accurate recordings of physical activity for each individual animal. Control animals remained sedentary.

*Supplementary Figure 2: Experimental setup.*

*
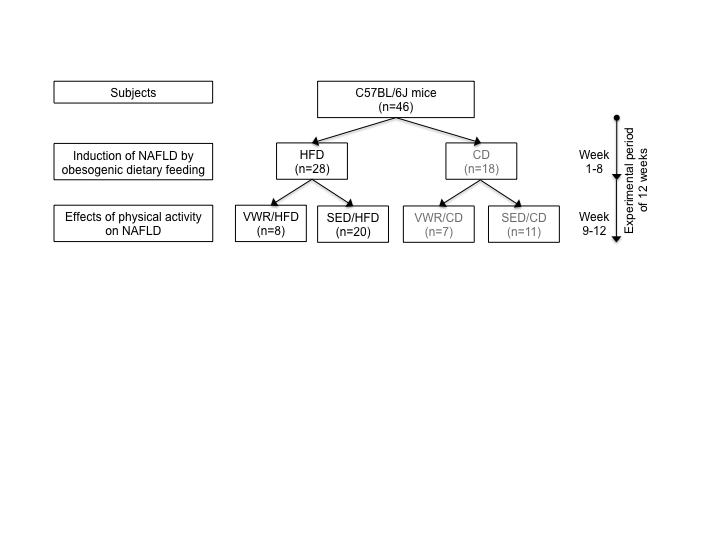
*

Male C57BL/6J mice (n=46), aged 8-10 weeks, were fed ad libitum either an obesogenic high-fat, high-carbohydrate diet (HFD, n=28) to induce NAFLD or a matched control diet (CD, n=18) for 12 weeks. Starting at week 9 of dietary feeding, mice were randomly selected to either voluntary wheel running (VWR, VWR/HFD n=8 and VWR/CD n=7) or to remain sedentary (SED, SED/HFD n=20 and SED/CD n=11) for a duration of 4 weeks while continuing HFD/CD-feeding. At the end of this training/sedentary period, VWR and SED mice were killed and blood and liver tissue were harvested to examine the impact of voluntary physical activity on NAFLD.

*Supplementary Figure 3: Immunoblots blots represented in main Figure 4.*

*
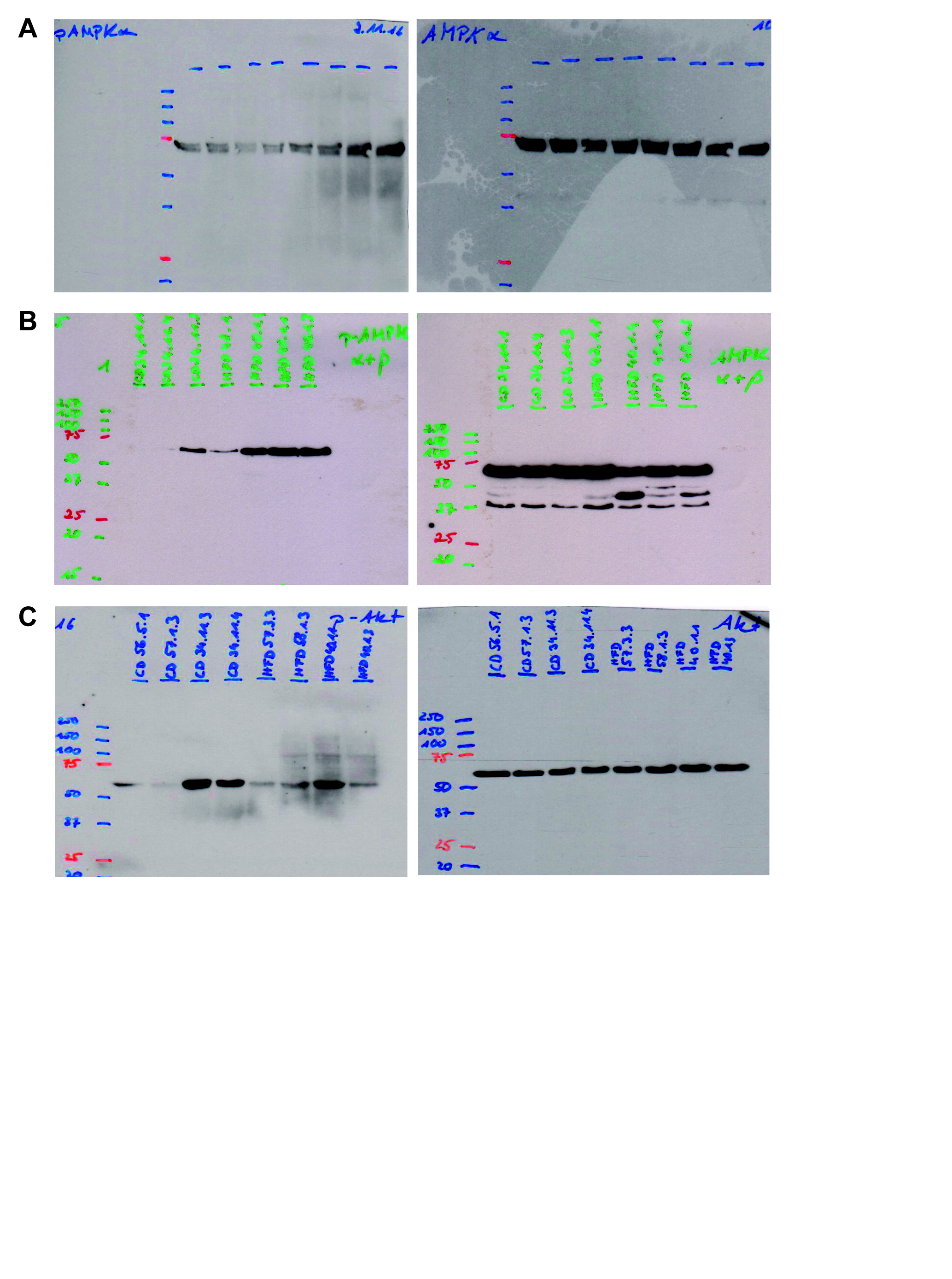
*

Chemiluminescent detection of the immunoblots shown in the manuscript in (A) Figure 4A, (B) Figure 4B and (C) Figure 4D.

*Supplementary Figure 4: Immunoblots represented in main Figure 6.*

*
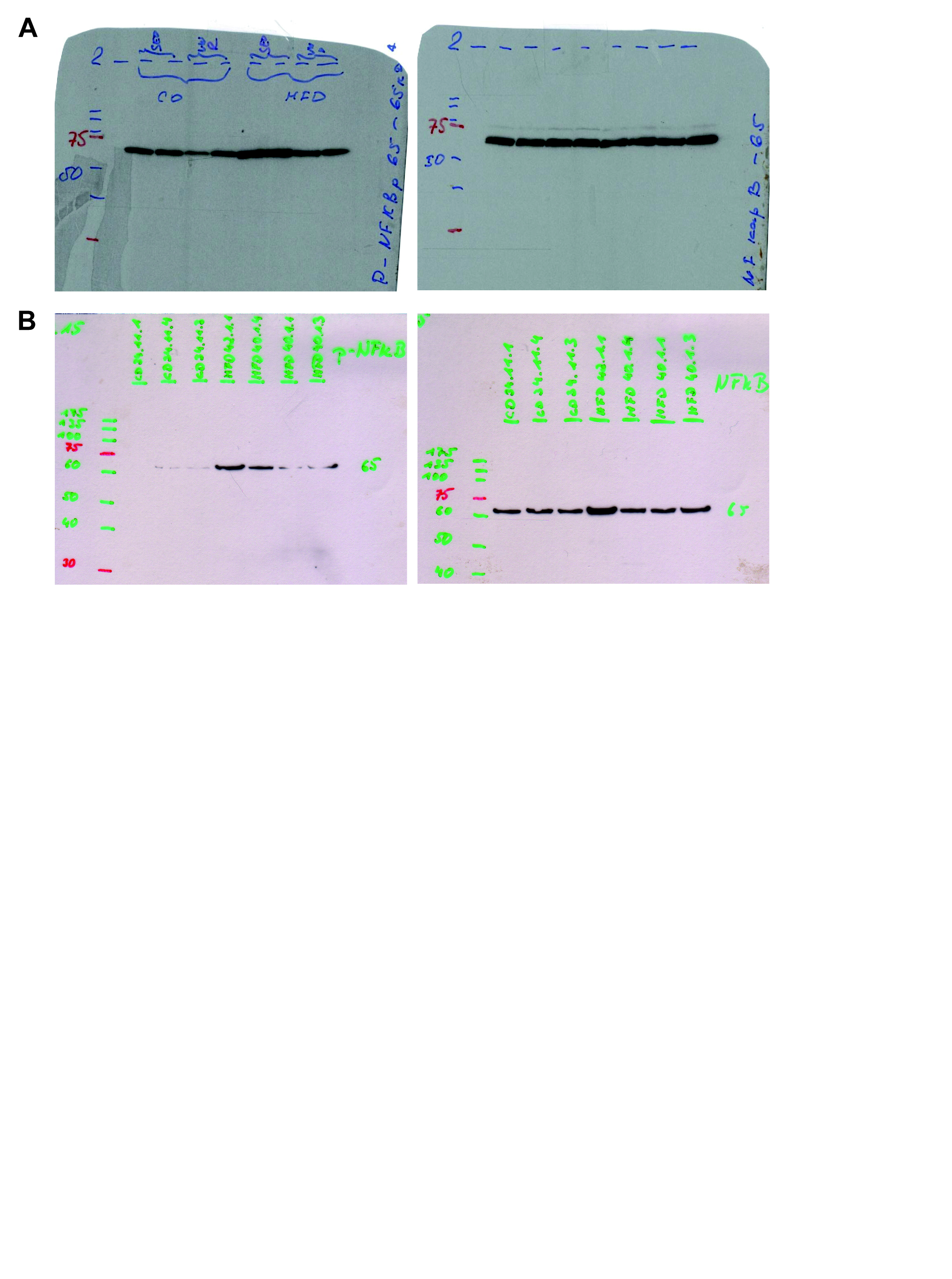
*

Chemiluminescent detection of the immunoblots shown in the manuscript in (A) Figure 6A and (B) Figure 6B.

*Supplementary Figure 5: Serum adiponectin and IL-6.*

*
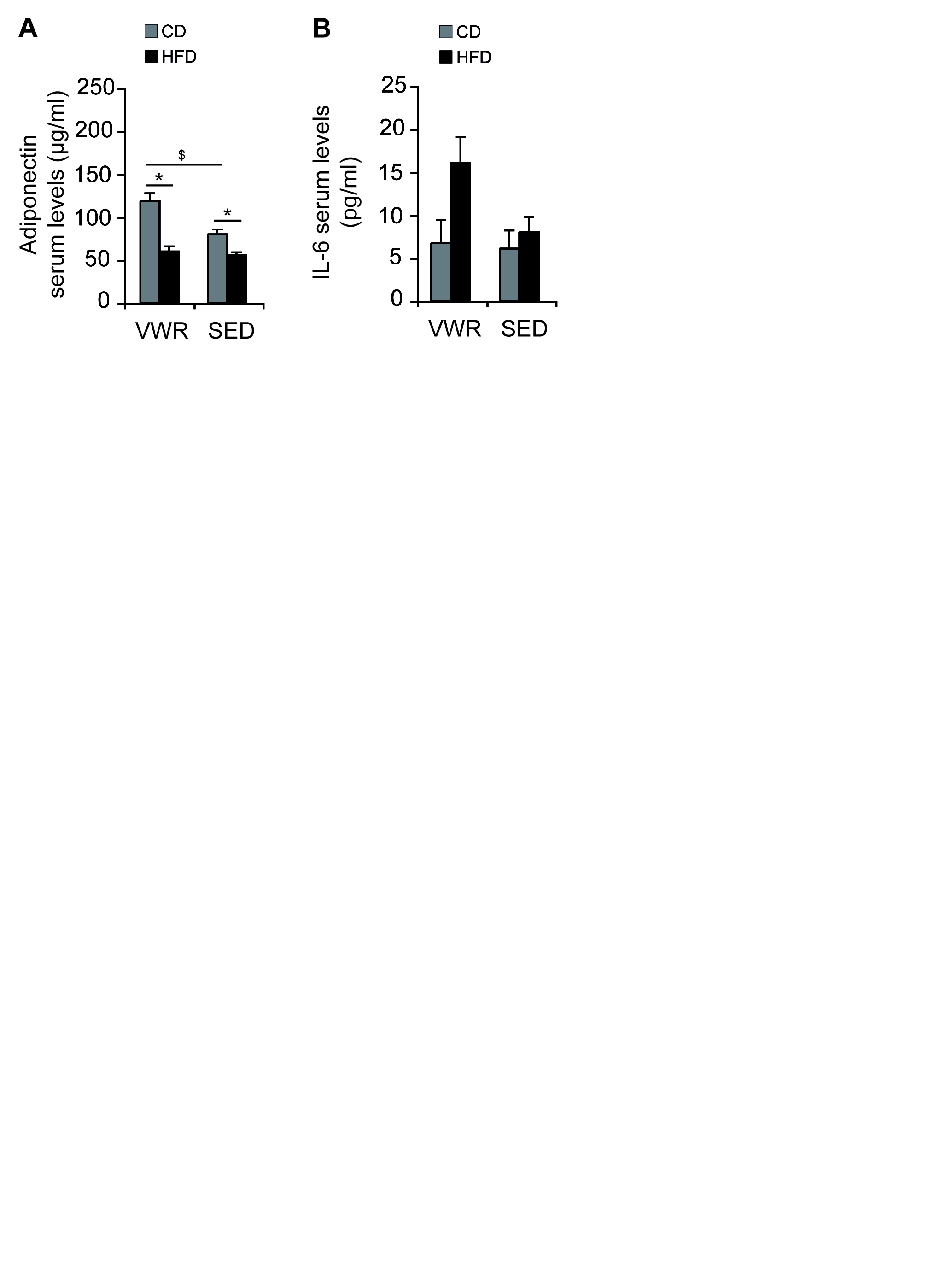
*

Analysis of (A) adiponectin and (B) IL-6 serum levels in VWR and SED mice after 12 weeks of exposure to CD or HFD. Determination was made by ELISA assays ((A) Thermo Fisher Scientific, Vienna, Austria, and (B) BioLegend, San Diego, CA, USA) according to the manufacturer’s protocol. Data in A and B are means ± SEM of n=4 VWR/CD, n=4 VWR/HFD, n=6 SED/CD, n=10 SED/HFD mice. *p<0.05 for CD vs. HFD and ^$^ p<0.05 for SED vs. VWR using two-tailed Student’s *t*-test (A).

**Supplementary Tables:**

*Supplementary Table 1: Crude nutrients and energy density of experimental diets (both ssniff Spezialdiäten GmbH, Soest, Germany).*

| **Product No.** |  | **Surwit Diet (HFD)**  **[HF/sucrose]**  **E15772-34** | **Control Diet (CD)**  **[soybean oil]**  **E15772-04** |
| --- | --- | --- | --- |
| Casein | % | 23.00 | 23.00 |
| Corn stach, pre-gelatinized | % | - | 27.00 |
| Maltodextrin | % | 15.30 | 15.30 |
| Sucrose | % | 17.00 | 17.00 |
| Pur. Cellulose powder | % | 1.80 | 5.20 |
| DL-Methionine | % | 0.10 | 0.10 |
| L-Cystine | % | 0.10 | 0.10 |
| Mineral & trace element premix | % | 5.50 | 5.50 |
| Vitamin premix | % | 1.00 | 1.00 |
| Sodium phosphate, dibasic | % | 0.30 | 0.30 |
| Dye, red-yellow mix | % | 0.10 | - |
| Choline chloride | % | 0.20 | 0.20 |
| Coconut oil, hydrogenated | % | 33.30 | - |
| Soybean oil | % | 2.30 | 5.30 |
| Metabolizable energy (ME), Atwater* | MJ/kg | 22.80 | 15.70 |
| Protein | kJ% | 15 | 22 |
| Carbohydrates | kJ% | 26 | 65 |
| Lipids | kJ% | 58 | 13 |

* physiological fuel value

*Supplementary Table 2:* *Forward and reverse primers used for qRT-PCR.*

| **Gene** | **Forward primer** | **Reverse primer** |
| --- | --- | --- |
| *Acaca* | AAC ATC CCC ACG CTA AAC AG | CTG ACA AGG TGG CGT GAA G |
| *Adgre1* | CTT TGG CTA TGG GCT TCT AGT C | GCA AGG AGG ACA GAG TTT ATC GTG |
| *Ccl2* | CTT CTG GGC CTG CTG TTC A | CCA GCC TAC TCA TTG GGA TCA |
| *Cpt1a* | AGT GGC CTC ACA GAC TCC AG | GCC ATG TTG TAC AGC TTC C |
| *Fasn* | CCC TTG ATG AAG AGG GAT CA | GAA CAA GGC GTT AGG GTT GA |
| *Fbp1* | ATG AGG GTT ATG CCA AGG ACT TT | CCA TCC GGA GGG AAC TTT TT |
| *G6pc* | TCC TGG GAC AGA CAC ACA AG | CAA CTT TAA TAT ACG CTA TTG G |
| *Il1b* | TCT TTG AAG TTG ACG GAC CC | TGA GTG ATA CTG CCT GCC TG |
| *Il6* | AGT TGC CTT CTT GGG ACT GA | TTC TGC AAG TGC ATC ATC GT |
| *Nr1h4* | CCC AGA GAA GAA CCG AGT T | TAG ATG CCA GGA GAA TAC CAG |
| *Pck1* | CTT CTC TGC CAA GGT CAT CC | TTT TGG GGA TGG GCA C |
| *Ppargc1a* | CGG AAA TCA TAT CCA ACC AG | TGA GGA CCG CTA GCA AGT TTG |
| *Ppara* | CAG TGG GGA GAG AGG ACA GA | AGT TCG GGA ACA AGA CGT TG |
| *Pparg* | GAT GGA AGA CCA CTC GCA TT | AAC CAT TGG GTC AGC TCT TG |
| *Srebf1* | ATC TCC TAG AGC GAG CGT TG | TAT TTA GCA ACT GCA GAT ATC CAA G |
| *Tgfb2* | TTC CTG GCG TTA CCT TGG T | CCA CTG CCG GAC AAC T |
